# Could Vagus Nerve Stimulation Target Hippocampal Hyperactivity to Improve Cognition in Schizophrenia?

**DOI:** 10.3389/fpsyt.2015.00043

**Published:** 2015-03-24

**Authors:** Jason Smucny, Adrienne Visani, Jason R. Tregellas

**Affiliations:** ^1^Neuroscience Program, University of Colorado Anschutz Medical Campus, Aurora, CO, USA; ^2^Research Service, Denver Veterans Affairs Medical Center, Denver, CO, USA; ^3^Department of Psychiatry, University of Colorado Anschutz Medical Campus, Aurora, CO, USA

**Keywords:** vagus nerve stimulation, schizophrenia patients, hippocampus, cognition, acetylcholine

Despite the fact that cognitive function is the best predictor of functional outcome and quality of life in schizophrenia (1), cognitive symptoms remain poorly treated in the illness. A myriad of cognitive domains are affected, including selective and sustained attention, working memory, episodic memory, processing speed, executive function, and social cognition (2). Patients consequently suffer from high unemployment rates (80%) and most are unable to live independently (30%) (3). Clearly, new treatments are needed.

One strategy for developing new interventions for these symptoms is to focus on treatments that may target biological indicators (“biomarkers”) of cognitive dysfunction. Recently, our laboratory found that resting-state hyperactivity of the hippocampus (as examined by functional magnetic resonance imaging) was strongly predictive of poor cognition in schizophrenia patients (4). This finding, along with previous studies that have demonstrated increased hippocampal blood flow (5), blood volume (6, 7), and hyperactivity during sensory processing (8, 9) in the disease suggests that hippocampal hyperactivity may be a biomarker for cognitive dysfunction in the illness (10). Loss of inhibitory signaling in the hippocampus is also hypothesized to play a role in sensory filtering deficits in schizophrenia (11), one of the most prominent electrophysiological features of the disease. This loss is thought to be conveyed through reduced nicotinic (12, 13) and/or GABAergic (14) signaling. It follows that interventions that reduce hippocampal hyperactivity may have therapeutic benefit in schizophrenia.

First developed in the 1980s for epilepsy, vagus nerve stimulation (VNS) may be a promising method for targeting hippocampal hyperactivity and improving cognition in schizophrenia. The vagus nerve (VN) is the longest cranial nerve, extending from the brain to the abdominal cavity. Although the VN is traditionally thought to primarily mediate central nervous system control of parasympathetic function, in actuality the VN consists of 80% afferent signals (from external organs to the brain) and 20% efferent signals (from the brain to organs) (15). VNS is most commonly achieved by surgical implantation of a stimulating, current-carrying wire around the nerve in the neck (16). The wire is intermittently stimulated by a battery-operated generator that is implanted in the left chest wall. Stimulation is directional (going to the brain), minimizing potential side effects from the VN’s efferent projections. Stimulus parameters (e.g., stimulus intensity, frequency, and duration) can be programed by a physician to maximize efficacy. More recently, a non-surgical method of VNS known as transcutaneous VNS (t-VNS) has been developed, in which the auricular branch of the VN is stimulated by electrodes placed on the outer ear (17–19). This stimulation site is in close proximity to an acupuncture site that may stimulate the VN in a similar manner (20). Interestingly, a number of studies have shown poor parasympathetic regulation of autonomic function in schizophrenia (21–26), suggesting that hypoactive VN function may contribute to disease pathophysiology.

By using VNS in combination with various functional neuroimaging techniques, researchers have frequently examined how VNS affects human brain activity. One of the most consistent neurophysiological effects of VNS is decreased hippocampal activity, possibly through enhancement of GABAergic signaling (27). As shown in Table 1, decreased hippocampal activity after VNS has been reported in over 15 studies (18, 28–44), although some others have not reported significant effects (45, 46). Remarkably, this effect has been observed in epileptic patients, depressed patients, and healthy subjects, suggesting that VNS may decrease hippocampal activity independent of the pathological state of the subject. Most relevant to schizophrenia, a recent *in vivo* electrophysiology study using the methylazoxymethanol acetate (MAM) rodent model of schizophrenia found decreased hippocampal hyperactivity in rats after 2 weeks VNS treatment (44). Furthermore, deep brain stimulation-induced reduction of hippocampal hyperactivity improves cognitive flexibility in the MAM rat model (47), suggesting that hyperactivity of the region may be targeted to improve cognition in schizophrenia.

**Table 1:** **Studies that have demonstrated effects of VNS on hippocampal activity**.

Subject population	VNS method	Imaging technique	Effect on HC rBF/activity	Reference
Epilepsy	s-VNS	PET	↓	Henry et al. (28)
		PET	↓	Henry et al. (29)
		HC depth	↓(epileptiform	Olejniczak et al. (33)
		electrodes	sharp waves)
		SPECT	↓	Vonck et al. (31)
		SPECT	↓	Van Laere et al. (30)
		SPECT	↓	Van Laere et al. (34)
		SPECT	↓	Barnes et al. (35)
		PET	↓	Henry et al. (36)
		SPECT	↓	Vonck et al. (41)
Depression	s-VNS	SPECT	↓	Devous (32)
		fMRI	↓	Mu et al. (37)
		SPECT	↓	Zobel et al. (39)
		PET	n.s.	Pardo et al. (45)
		PET	n.s.	Conway et al. (46)
Healthy	t-VNS	fMRI	↓	Kraus et al. (40)
		fMRI	↓	Kraus et al. (42)
		fMRI	↓	Frangos et al. (43)
Animal (rat)	s-VNS	PET	↓	Dedeurwaerdere et al. (38)
		*in vivo* electrophysiology	↓	Perez et al. (44)

*fMRI, functional magnetic resonance imaging; HC, hippocampus; n.s., no significant effects; PET, positron emission tomography; rBF, regional blood flow; s-VNS, surgical VNS; SPECT, single-photon emission computed tomography; t-VNS, transcutaneous (non-surgical) VNS*.

Vagus nerve stimulation also has demonstrated pro-cognitive effects in a number of studies. As reviewed by Vonck et al. (48), improved cognition after VNS has been observed in patients with epilepsy, Alzheimer’s, and depression. Cognitive domains that have shown improvement include verbal recognition, attention, memory consolidation, and executive function (48). Other studies, however, have not shown significant effects, possibly due to changes in stimulus or study parameters (e.g., placebo design) (48). Indeed, studies that examine the effects of chronic VNS often lack appropriate placebo controls due to ethical issues in applying “placebo” stimulation protocols to patient populations. This concern may be addressed by studies in healthy subjects using non-invasive t-VNS, in which placebo “stimulation” is delivered by an electrode placed on the earlobe (27). Nonetheless, the effects of non-invasive t-VNS on cognitive performance have yet to be well evaluated.

The effects of VNS on neurotransmitter systems show important parallels with current lines of therapeutic investigation in schizophrenia. Ascending projections of the VN terminate in the nucleus tractus solitarius in the medulla, which in turn innervates the locus coeruleus and induces the release of norepinephrine (NE) (49, 50). Activation of adrenergic receptors in the hippocampus can thereby reduce the excitability of the region (51–53). In line with a role for increased adrenergic signaling in the treatment of schizophrenia, adrenergic α2 receptor agonists such as clonidine are currently being investigated in early phase trials in patients. Preliminary studies have demonstrated that activation of this receptor improves sensorimotor (54) and auditory gating (55) deficits in the disease. Guanfacine, an α2A receptor-specific agonist, also shows pro-cognitive effects in several patient populations including schizophrenia (56–58).

A secondary effect of VNS directly relevant to schizophrenia is to increase cholinergic neurotransmission by NE-induced activation of postsynaptic beta and α1 adrenergic receptors in the basal forebrain, leading to increased acetylcholine (ACh) release (59). This increase may help normalize deficits in cholinergic neurotransmission in the disorder driven in large part by a significant reduction in the expression of nicotinic ACh receptors on inhibitory interneurons (11, 12, 60–63). Loss of these receptors is also hypothesized to contribute to hippocampal hyperactivity (10, 11, 62), which may be normalized by nicotinic activation of receptors on inhibitory interneurons (61, 64). In support of this view, nicotine, a nicotinic receptor agonist, reduces hippocampal hyperactivity observed during smooth pursuit eye movement in schizophrenia patients (65, 66). This attempt to “self-medicate” may help explain why the majority of patients smoke cigarettes (67). VNS may be a particularly effective method of potentiating nicotinic signaling as its cholinergic effects are conveyed via increased release of ACh. Relative to other nicotinic agonists (such as nicotine), ACh is more quickly degraded by endogenous enzymes, and is therefore less likely to induce receptor desensitization (68).

A third mechanism by which VNS may affect the neurobiology of schizophrenia is via its ability to increase serotonin release (69). Increased serotonergic tone may in turn decrease hippocampal hyperexcitability by potentiating GABAergic signaling through activation of several serotonin receptor subtypes, including 5-HT-2, 3, and 4 (70–73). Activation of the 5-HT1 serotonin receptor subtype is also associated with hippocampal neurogenesis and has been proposed as a potential mechanism to restore pattern separation (a memory-related hippocampal function) deficits in schizophrenia (74). Furthermore, activation of 5-HT3 receptors may relieve tonic inhibition of ACh release (75), increasing cholinergic tone and restoring hippocampal nicotinic signaling in the illness. How VNS specifically affects activity at these receptor targets and their relationship to cognition and hippocampal activity are important areas for future research.

Vagus nerve stimulation, whether administered by surgical implant or transcutaneous stimulation, is a well-tolerated procedure. Risks associated with the surgery itself are minimal (76). The most common side effects associated with stimulation are hoarseness, dyspnea, and cough. Most of these effects decrease with time (77). Nonetheless, VNS may have more significant risks specific to schizophrenia. Compounds that non-specifically (across all adrenergic receptor subtypes) increase noradrenergic activity, such as amphetamine, are known to induce psychosis in normal subjects (78, 79) and worsen positive symptoms in patients (80). Indeed, VNS itself has been associated with the onset of psychosis in epileptic patients in a limited number of case studies (81). It is possible that schizophrenia patients who are taking antipsychotic medication that participate in a VNS study will require adjustment of antipsychotic doses in order to preserve clinical efficacy. Previous studies, however, have also observed positive correlations between VN/parasympathetic nervous system dysfunction and positive symptoms, suggesting that VNS may actually improve these symptoms (24, 25). As the interactions between VNS and antipsychotic efficacy in schizophrenia are entirely unknown, studies should make closely monitor core symptoms of the illness associated with acute and chronic VNS treatment.

Despite its potential to improve cognition in schizophrenia, to our knowledge no study has yet examined the clinical or physiological effects of VNS in patients with the disease. Lack of research in this area may be due in large part to the invasiveness of traditional, surgical VNS. To that end, the advent of t-VNS provides a simple, non-invasive method for examining the acute and chronic effects of stimulation in schizophrenia under various stimulus protocols. The recent discovery of hippocampal hyperactivity as a biomarker for cognitive symptoms in schizophrenia provides a useful mechanism to establish target validation using functional neuroimaging. In regards to VNS, the fact that numerous studies have repeatedly demonstrated that stimulation reduces hippocampal activity in other patient populations suggests that it may have the same effect in schizophrenia. Furthermore, although this article has focused on hippocampal effects, VNS may also improve cognition through other mechanisms, such as neurochemical modulation of the prefrontal cortex (82–84) and/or prefrontal–hippocampal interactions (85). In summary, examination of the potential effects of VNS for cognitive symptoms in schizophrenia may be a promising future research direction.

## Conflict of Interest Statement

The authors declare that the research was conducted in the absence of any commercial or financial relationships that could be construed as a potential conflict of interest.
